# Fertilization in the African clawed frog *, Xenopus laevis* , requires an egg-derived PLC to signal the fast block to polyspermy

**DOI:** 10.17912/micropub.biology.001638

**Published:** 2025-07-30

**Authors:** Kayla M. Komondor, Katherine G. Sharp, Anne E. Carlson

**Affiliations:** 1 Biological Sciences, University of Pittsburgh, Pittsburgh, Pennsylvania, United States

## Abstract

Fertilization in *Xenopus laevis* triggers calcium release and membrane depolarization to activate the fast block to polyspermy. This depolarization requires phospholipase C (PLC) activity, but the identity and origin of the PLC remained unclear. We previously reported that *PLCZ1* , which encodes the sperm-derived PLCζ used in mammals and birds, was deleted in Pipidae frogs, suggesting an alternative mechanism. Here, we used the irreversible PLC inhibitor U73122 to test whether the PLC driving the fast block originates from the egg or sperm. Inhibiting PLC activity in the egg, but not in the sperm, abolished the fertilization-evoked depolarization and led to polyspermy. These findings demonstrate that *X. laevis* relies on an egg-derived PLC to signal the fast block, revealing a fertilization-triggered calcium signaling pathway that is mechanistically distinct from other vertebrates.

**Figure 1. An egg-derived PLC signals the fast block to polyspermy:**
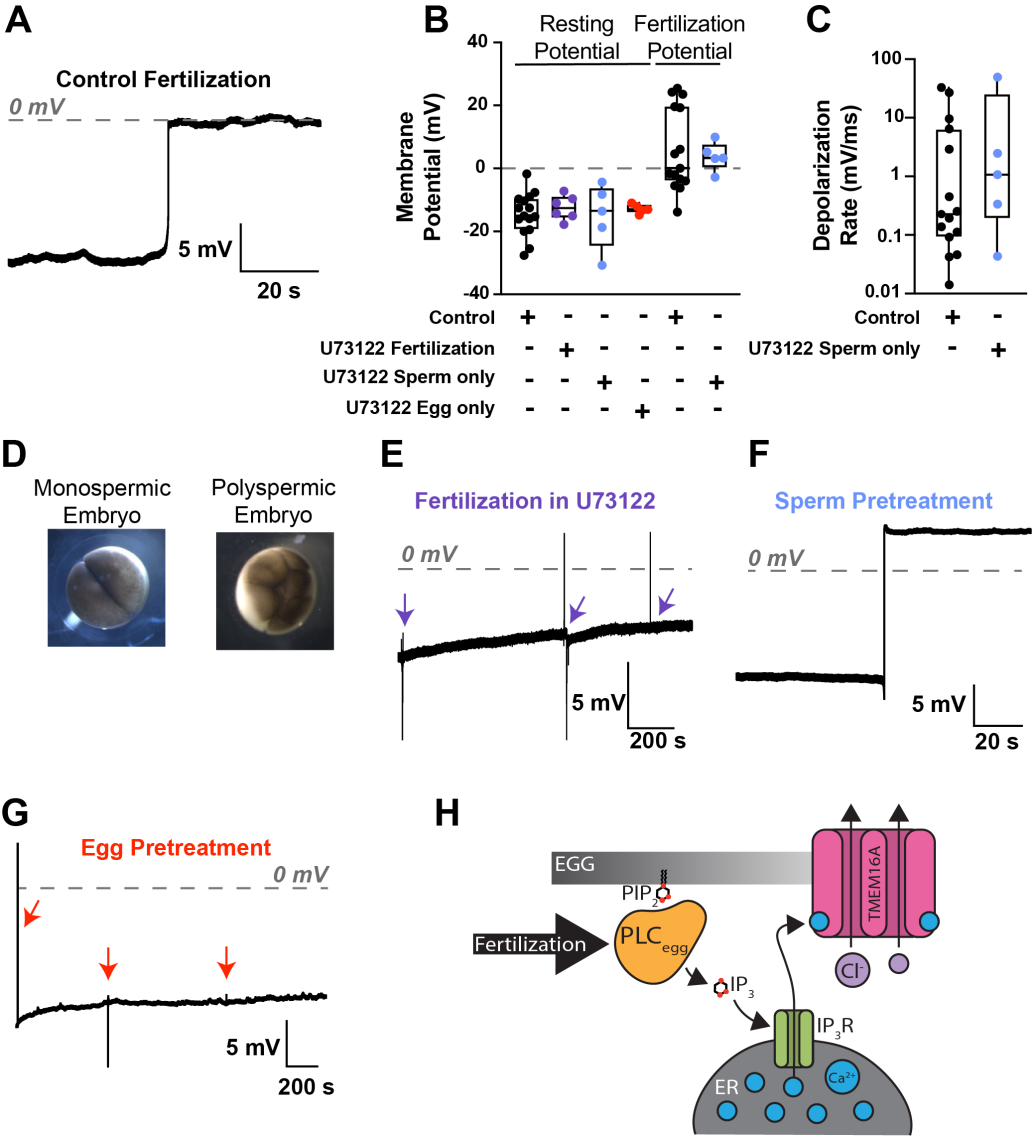
**(A)** Representative whole-cell recording during fertilization of NF stage 1 eggs, under control conditions. The grey dashed line denotes 0 mV. **(B)** Box plot distribution of the resting membrane potential and the fertilization potential of eggs made in the indicated conditions: Control, fertilization in 1 μM U73122, and from fertilization with sperm or eggs pretreated with 1 μM U73122 and fertilized under control conditions. The middle line denotes the median value, the box marks 25-75%, and the whiskers denote 0-100% (N=5-15). **(C)** Box plot distribution of the rate of fertilization signaled depolarization for recordings made in control conditions or with sperm pretreated with 1 μM U73122. **(D)** Representative images of NF stage 2 embryos from monospermic or polyspermic fertilizations. **(E-G)** Representative whole-cell recordings during fertilization in various conditions: in 1 μM U73122 **(E)** , and from fertilization with sperm **(F)** or eggs **(G)** pretreated with 1 μM U73122 and fertilized under control conditions. Arrows denote sperm addition. **(H)** Schematic of the experimentally established, fertilization-triggered and PLC-mediated signaling pathway that signals the fast block to polyspermy in *Xenopus laevis* eggs.

## Description

Fertilization triggers a rapid rise in cytoplasmic calcium in the eggs of most animals. This calcium then initiates the polyspermy blocks and the onset of embryonic development (Whitaker, 2006; Bainbridge & Carlson, 2023). In mammals and birds, calcium release is signaled by the sperm-derived enzyme phospholipase C zeta (PLCζ), which cleaves phosphatidylinositol 4,5-bisphosphate (PIP _2_ ) to generate inositol trisphosphate (IP _3_ ). IP _3_ then promotes calcium release from the endoplasmic reticulum (ER) (Saunders *et al.* , 2002; Swann & Lai, 2016).

In the African clawed frog, *Xenopus laevis* , fertilization signals PLC-dependent calcium release from the ER, which activates the calcium-activated chloride channel TMEM16A (Wozniak *et al.* , 2018a). The resulting chloride efflux depolarizes the egg membrane, producing the fast block to polyspermy (Wozniak & Carlson, 2020). We previously demonstrated that PLC activity is required for this depolarization (Wozniak *et al.* , 2018b). However, the identity and origin of the PLC responsible for this signaling remained unclear. Importantly, we also reported that the PLCZ1 gene, which encodes PLCζ, was deleted from the ancestral lineage of Pipidae frogs, including *X. laevis* (Bainbridge *et al.* , 2025). This evolutionary loss indicates that *X. laevis* sperm lack PLCζ and therefore cannot use the same sperm-derived mechanism. These findings prompted the question of whether the PLC that signals the fast block in *X. laevis* fertilization is a sperm-derived PLC other than PLCζ, or an egg PLC?

To distinguish between these possibilities, we took advantage of the maleimide moiety on the general PLC inhibitor U73122, which forms covalent bonds with cysteines in the PLC active site (Bleasdale *et al.* , 1990). This covalent modification results in irreversible inhibition of the enzyme. Here, we independently pretreated sperm or eggs with U73122 to determine which gamete provides the PLC responsible for the fast block in *X. laevis* fertilization.

To study the fast block to polyspermy, we use whole-cell recordings on *X. laevis* eggs during fertilization. Under control conditions, fertilization triggered a depolarization of the egg – the fast block to polyspermy – several minutes after sperm addition ( Figure 1A ). Specifically, fertilization shifted the membrane potential from an average resting value of -14.5 ± 1.8 mV to a fertilization potential of 5.8 ± 3.5 mV ( Figure 1B ), with an average rate of depolarization of 5.3 ± 2.8 mV/ms ( Figure 1C ) (N=15). All embryos developed symmetric cleavage furrows ( Figure 1D ), consistent with monospermic fertilization (Elinson, 1975). By contrast, fertilization never triggered a depolarization of eggs inseminated in the presence of 1 μM of the broad PLC inhibitor U73122 ( Figure 1E ). All five eggs inseminated in U73122 initiated development, but the embryos cleaved with asymmetric furrows ( Figure 1D ), consistent with fertilization by more than one sperm. Together, these data reveal that fertilization occurred, but the membrane depolarization was stopped.

To investigate whether the required PLC for the fast block comes from the sperm, we pretreated *X. laevis* sperm with 1 μM U73122 for 10 minutes before insemination. Fertilization evoked normal depolarizations when U73122-pretreated sperm were applied to untreated eggs ( Figure 1F ). Eggs displayed resting potentials (-15.1 ± 4.1 mV) and fertilization potentials (3.8 ± 1.8 mV) that were not different from control conditions (-13.4 ± 1.0 mV and 3.2 ± 1.8 mV, *respectively* ) ( Figure 1B ). Similarly, the depolarization rate (10.6 ± 8.6 mV/ms, N = 5) did not differ from control recordings (6.7 ± 1.9 mV/ms) ( Figure 1C ). All embryos developed symmetric cleavage furrows, consistent with fertilization by a single sperm. This result suggests that a sperm-derived PLC does not signal the fast block in *X. laevis* fertilization.

By contrast, when eggs were pretreated with 1 μM U73122 for 10 minutes and then inseminated without the inhibitor, fertilization did not trigger a depolarization ( Figure 1G ). Resting potentials in pretreated eggs (-12.8 ± 0.6 mV, N = 5) were not different from controls. Moreover, U73122-pretreated eggs developed asymmetric furrows ( Figure 1D ), consistent with polyspermic fertilization. Together, these results indicate that the fast block to polyspermy requires activation of an egg-derived PLC.

Altogether, our findings demonstrate that the fertilization-triggered depolarization in *X. laevis* requires PLC activity and that the PLC is provided by the egg rather than the sperm. This mechanism contrasts with fertilization strategies in mammals, birds, and other amphibians, where sperm-derived PLCζ initiates calcium signaling. Our results rely on U73122, which is the best available inhibitor for PLC activity; however, this compound may have off-target effects, including inhibition of adenosine A1 receptors (Walker et al., 1998).

Previous work has suggested that an egg-derived PLC contributes to the calcium signaling during fertilization in *X. laevis* . A published model proposes that fertilization in *X. laevis* activates PLCγ1 through a tyrosine kinase-dependent signaling pathway (Sato *et al.* , 2000). PLCγ enzymes are typically activated by phosphorylation of a critical tyrosine residue near the catalytic domain (Gresset *et al.* , 2010). However, we have found that this critical PLCγ1 tyrosine is not phosphorylated during fertilization of *X. laevis* eggs, or that tyrosine kinase activity is not required for the fast block (Komondor *et al.* , 2023). Together, these findings suggest that the fast block to polyspermy is not mediated by a typical tyrosine kinase-dependent activation of PLCγ1.

Combined with our previous data, these results suggest that fertilization activates an egg PLC to trigger the fast block. Identifying how fertilization engages an egg PLC remains a key next step toward understanding calcium signaling during fertilization in Pipidae frogs.

## Methods

All procedures were approved by the Institutional Animal Care and Use Committee at the University of Pittsburgh and conducted in accordance with established standards of humane animal care. Adult *Xenopus laevis* were purchased from Xenopus 1 (Dexter, MI) and housed at 18°C on a 12/12 hr light/dark cycle in dechlorinated municipal water buffered with sodium bicarbonate.

Females were induced to ovulate by injection of 1,000 IU of human chorionic gonadotropin (Chorulon) into the dorsal lymph sac, followed by incubation at 14–16°C for 12–16 h. Females typically began laying eggs within 0–2 hours of transfer to room temperature (22°C). Eggs were collected onto dry Petri dishes and used within 10 min of laying. Testes were harvested from adult males euthanized by a 30-min immersion in 3.6 g/L tricaine-S (MS-222, pH 7.4) and stored at 4°C in L-15 medium for use within 5 days.

Fertilization-competent sperm suspensions were generated by mincing 1/10 of a testis in modified Ringer’s solution diluted 1:5 (MR/5) for electrophysiology. Modified Ringer’s (MR) contained (in mM): 100 NaCl, 1.8 KCl, 2.0 CaCl₂, 1.0 MgCl₂, and 5.0 HEPES, pH 7.8, and was filtered through a sterile 0.2-μm polystyrene filter. Eggs were inseminated by pipetting sperm suspension over the eggs in MR/5 (for recording).

Electrophysiological recordings were made in whole-cell configuration using TEV-200A amplifiers (Dagan Co.) and digitized using Axon Digidata 1550A (Molecular Devices). Recordings were collected with pClamp Software (Molecular Devices) at 5 kHz. Pipettes were pulled from borosilicate glass to a resistance of 8–20 MΩ and filled with 1 M KCl. Resting and fertilization membrane potentials were quantified ~10 s before and after the depolarization, respectively. Depolarization rates were calculated by measuring the slope of the fastest 1-mV shift in membrane potential. Recordings were analyzed using IGOR (WaveMetrics), Excel (Microsoft), and Prism (GraphPad).

To assess whether fertilization-evoked depolarization requires phospholipase C (PLC), we used the irreversible PLC inhibitor U73122 (Cayman Chemical). U73122 was prepared in DMSO and diluted to 1 μM in MR/5. The final DMSO concentration was maintained below 2%, which does not interfere with fertilization or depolarization (Wozniak *et al.* , 2018a). In some experiments, either eggs or sperm were pretreated with 1 μM U73122 for 10 minutes, washed in fresh MR/5, and inseminated in inhibitor-free solution.

To assess polyspermy, inseminated eggs were transferred to MR/3 and allowed to develop at room temperature for 2-3 h. Embryos were evaluated at the 4-cell (NF stage 3) for cleavage furrow symmetry. Monospermic embryos developed symmetric furrows, whereas polyspermic embryos displayed asymmetric or incomplete furrows.

Depolarization rates were log _10_ -transformed prior to analysis. All comparisons were made using one-way ANOVA followed by Tukey’s HSD test in Prism (GraphPad). All experiments were repeated on at least three independent days using gametes from different animals and conducted at 22°C.

## Reagents

**Table:** 

**Reagent**	**Source**	**Catalog Number / Identifier**
*Xenopus laevis*	Xenopus 1, Dexter MI	Wild-type strains: oocyte-positive females and adult males
Chorionic gonadotropin (Chorulon)	Covetrus	Cat. No. 28938 UniProt P01215
Tricaine-S (MS-222)	Thermo Fisher	Cat. No. NC0342409
L-15 Medium (without L-glutamine)	Thermo Fisher	Cat. No. L1518-500ml
Phospholipase C inhibitor U73122	Cayman Chemical	Cat. No. 70740 CAS No. 112648-68-7 PubChem CID: 104794
DMSO	Thermo Fisher	CAS No. 67-68-5
Modified Ringer’s Salts	Thermo Fisher or Sigma	Standard lab-grade reagents (NaCl, KCl, CaCl₂, MgCl₂, HEPES)
